# Investigating the genomic alteration improved the clinical outcome of aged patients with lung carcinoma

**DOI:** 10.1186/s12864-021-08289-4

**Published:** 2022-01-15

**Authors:** Sixian Chen, Aizhen Fu, Yuan Lu, Wei Lu, Yongfeng Chen, Shuiqiang Hong, Suli Zhou, Tianmin Xiang, Zhenzhen Zhang, Yongguang Cai

**Affiliations:** 1 Medical Oncology Department V, Guangdong Nongken Central Hospital, Zhanjiang, 524002 China; 2 Gynecology Department, Affiliated Hospital of Guangdong Medical University, Zhanjiang, 524001 China; 3 Singlera Genomics Inc., Shanghai, 201318 China

**Keywords:** Lung cancer, Genomic alterations, *KRAS* codon 61, Targeted treatment, Mechanism of resistance

## Abstract

**Background:**

Lung carcinoma is a common geriatric disease. The development of genotype-targeted therapies greatly improved the management of lung carcinoma. However, the treatment for old patients can be more complex than that for young individuals.

**Results:**

To investigate the benefits of genetic detection for older patients with lung carcinoma, we explored the genomic profiling of 258 patients with more than 55 years using a targeted next generation sequencing, and some of these patients were treated with targeted therapies based on the results of genomic detection. *KRAS* codon 61 mutations were found in 15.2% *KRAS*-mutated patients, which tend to be co-existing with other classical activating mutations other than codons 12/13. Acquired *EGFR* C797S mutations were identified in 2 cases and *ERBB*2 amplification was identified in 1 case. All these 3 cases developed resistance to EGFR tyrosine kinase inhibitors and showed expected results of their followed therapies. The median progression-free survival and median overall survival of patients treated with molecular targeted therapies were better than those of patients treated with chemoradiotherapy alone.

**Conclusions:**

Our findings revealed the specific genomic profiles of patients older than 55 years with lung carcinoma and suggested that these old patients have been benefit from the genetic detection, which helped identify druggable mutations and distinguish resistance mechanisms.

**Supplementary Information:**

The online version contains supplementary material available at 10.1186/s12864-021-08289-4.

## Background

Lung cancer, one of the most common malignancies worldwide, is the leading cause of cancer mortality [1]. The incidence of lung cancer is relatively low (1–10%) in the young adult patients [2], and increases with age [3]. Due to the aging trend observed in societies and the increasing availability of high-resolution computed tomography (CT) and positron emission tomography (PET), the incidence of lung cancer in older patients is markedly increasing.

The treatment of older patients with lung cancer can be more complex than that of younger patients due to the age-related decline in organ function, multiple comorbidities, concurrent medications, and possible presence of a geriatric syndrome [4, 5], which have the potential to increase drug-related toxicity and affect the ability of older patients to tolerate and continue treatment. The challenge for clinicians is how to select a treatment strategy for older patients that prolongation of survival time while maintaining quality of life.

Despite studies have showed that older patients with minimal comorbid conditions tolerated and respond to chemo just as well as young patients, older patients are not prescribed standard chemotherapy [6]. In recent years, many lung cancer patients have benefited from the personalized treatment like targeted therapy on the basis of the genetic background of the tumor [7]. *EGFR* mutations, *ALK* rearrangements and *ROS1* rearrangements are regarded as “must test” biomarkers in the molecular diagnosis of advanced lung carcinoma patients [8]. For the development of genotype-targeted therapies, it is necessary to identity oncogenic molecular changes leading to aberrant activation of intracellular signaling associated with the sustained growth of lung cancer cells. Other oncogenic alterations have been revealed like *MET*, *BRAF* and *HER2* as novel targets for personalized therapies [9, 10] by the next-generation sequencing (NGS) technology. Considering the high incidence of lung cancer in the older population, comprehensive information regarding specific molecular abnormalities is valuable in choosing feasible treatment options to maximize therapeutic benefits and minimize therapy-associated risks.

In the present study, a well-validated 12 gene panel for genomic analysis was applied to 258 Chinese lung carcinoma patients with more than 55 years old. Their genomic alterations were used to help designing the following medical treatment. The main objective of this study was to investigate the molecular characteristics of these old patients with lung carcinoma in China, and to find out whether these old patients were benefited from the genetic detection.

## Results

### Patient characteristics

The clinical features of all 258 Chinese patients with lung carcinoma in the cohort are summarized in Table 1 with details in Additional file 1: Table S1. The median age of patients at the time of first sampling was 69 years (range 55 to 99 years). Among these patients, 202 (78.3%) were classified as stage IV, and 44 (17.1%) were as stage I, II and III. For all the 258 patients enrolled, 249 were sampled once, while other 9 patients were sampled both before and after EGFR tyrosine kinase inhibitors (TKIs) treatment (Table 2).

**Table 1:** The baseline characteristics of 258 patients with lung carcinoma

Variables	*n* = 258 patients	%
Age (year)		
Median (range)	69 (55–99)	
Gender		
Male	135	52.3%
Female	123	47.7%
Smoking status		
Smokers	90	34.9%
Never smoked	160	62.0%
Unknown	8	3.1%
Histologic types		
Adenocarcinoma (ADC)	172	66.7%
Squamous cell carcinoma (SCC)	20	7.8%
Adenosquamous carcinoma (ASC)	3	1.2%
Large cell carcinoma	1	0.4%
Small-cell carcinoma	3	1.2%
Unknown	59	22.9%
Clinical stage		
I	5	1.9%
II	4	1.6%
III	35	13.6%
IV	202	78.3%
Unknown	12	4.7%

**Table 2:** The number of patients from which samples were collected

	No. of patients		
	FFPE	Plasma	Both
Sampled before treatment	103	103	–
Sampled after treatment	8	35	–
Targeted therapy	7	22	–
Targeted plus Chemotherapy	–	3	–
Chemotherapy	1	10	–
Sampled both before and after treatment	2	4	3^a^

^a^For the 3 patients, formalin-fixed paraffin-embedded (FFPE) samples were collected before treatment and plasma samples were collected after treatment

### Molecular profiling of lung carcinoma in aged patients

**Fig. 1:**
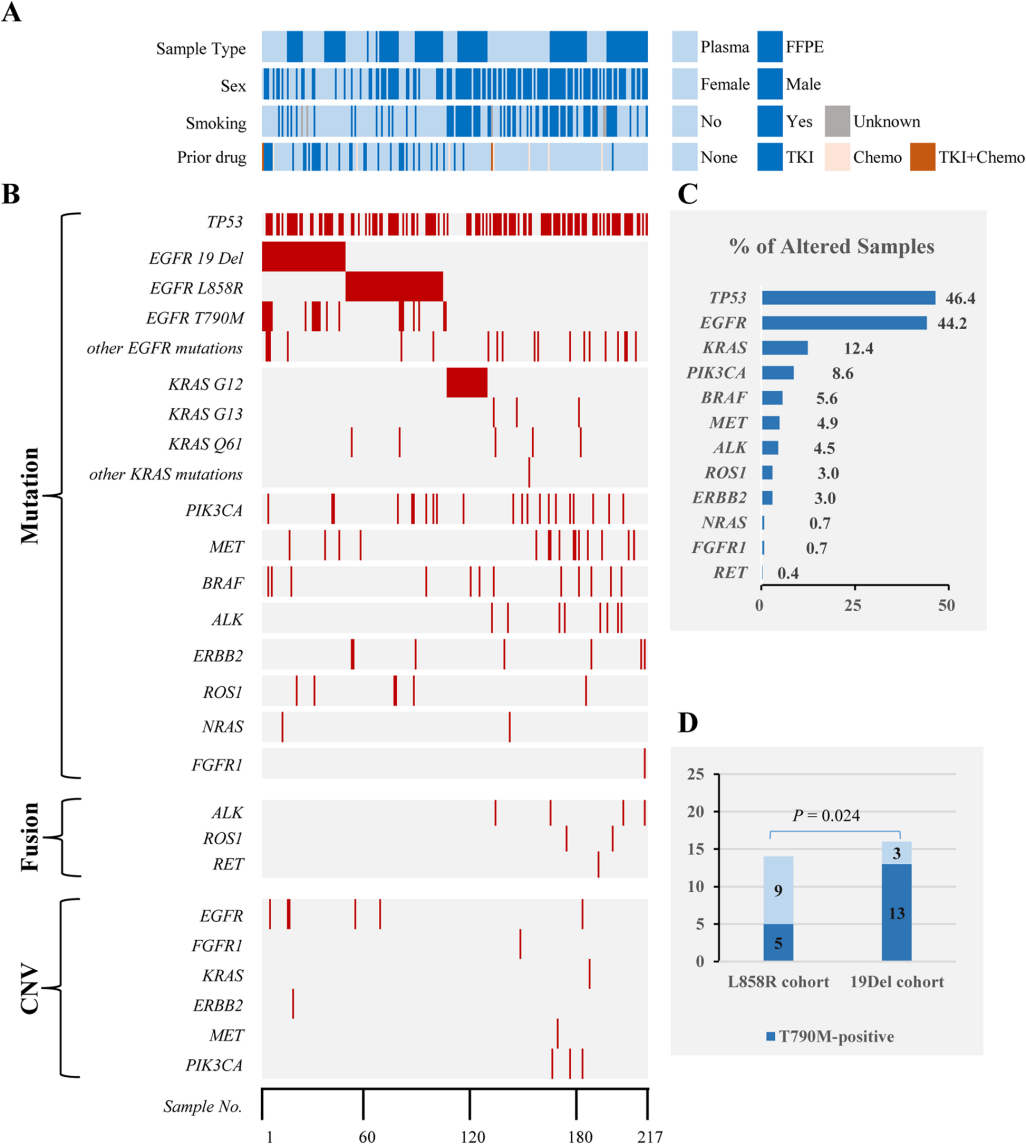
Landscape of genetic alterations of 217 lung carcinoma samples. **A** The baseline characteristics of gene-mutated lung carcinoma patients. Prior drug: drug history before sampling. **B** The distribution of genetic alterations identified in each specimen. **C** The number of samples having a certain alteration. **D** The number of T790M mutation in *EGFR* L858R or 19Del mutated patients who received 1st generation EGFR TKIs treatment before sampling

**Fig. 2:**
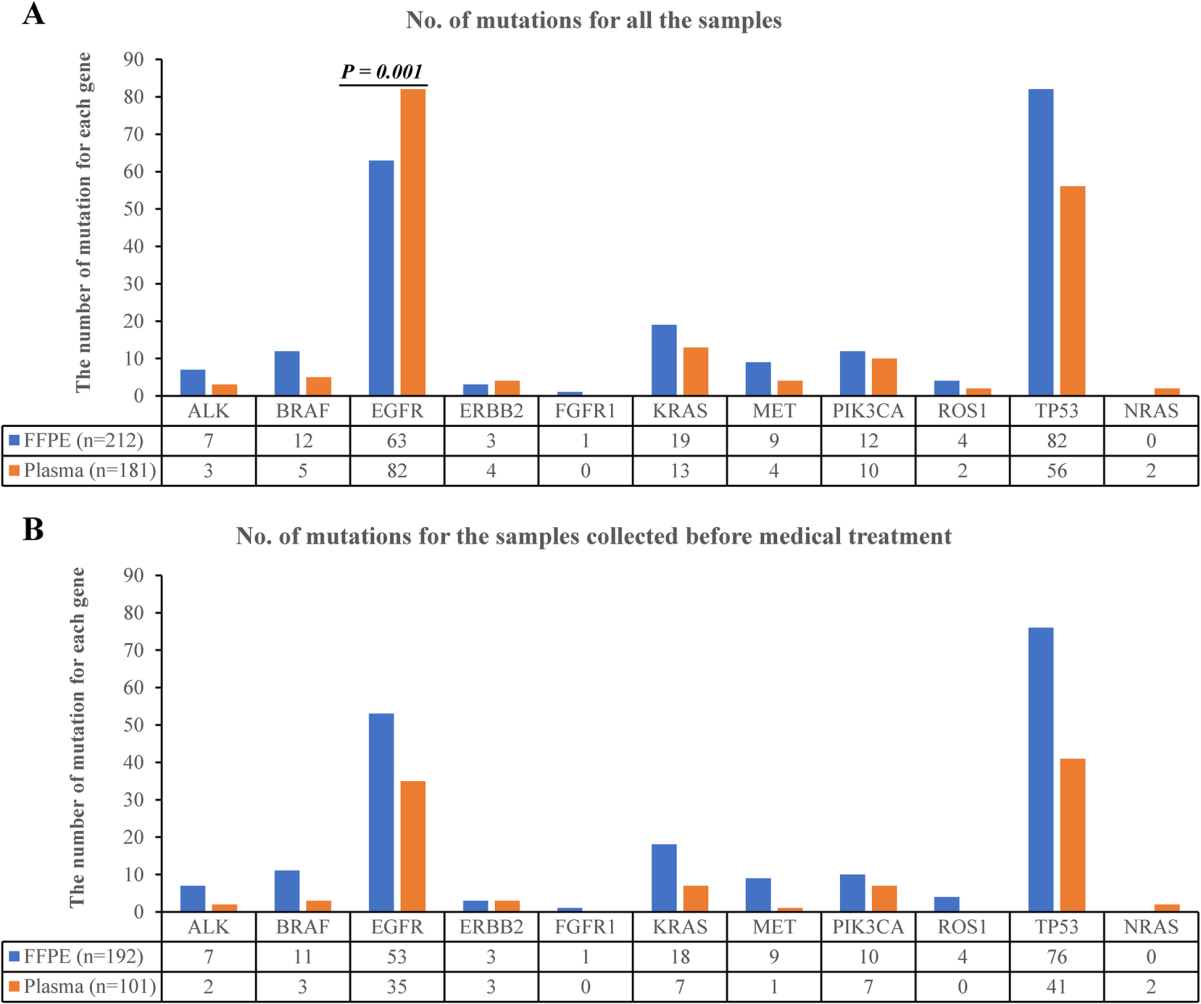
Distribution of gene mutations in FFPE tissue and plasma

Mutation analysis revealed that 217 samples harbored at least one gene alteration (Fig. 1). As shown in Fig. 1, the most frequently mutated genes *TP53* (46.4%, 124/267) and *EGFR* (44.2%, 118/267) were identified in about half of our cohort. *KRAS* mutations were found in 33 (12.4%, 33/267) patients. Other genes were mutated in no more than 10% of the patients enrolled. Compared with the TCGA population (https://portal.gdc.cancer.gov), we found a more mutation frequency of *EGFR* (53.9% vs. 14.6%, *P* < 0.001) and a less frequency of *KRAS* (15.2% vs. 27.9%; *P* < 0.001) in our cohort. Besides, *EGFR* mutation was more frequently observed in plasma samples compared to FFPE tissue specimens (*P* = 0.001). The most discrimination observed was the number of *EGFR* T790M (3 in FFPE and 18 in plasma samples). When we excluded those samples collected after medical treatment, no statistical difference of gene alteration was observed between the FFPE and plasma samples (Fig. 2). In addition, no difference was observed in specific mutational signature of single nucleotide variants (SNVs) between the FFPE and plasma samples for either all patients enrolled or therapy- naïve patients (Additional file 2: Fig. S1).

### *EGFR* mutations

*EGFR* exon 19 deletions (47/267, 17.6%) and exon 21 p.L858R mutation (55/267, 20.6%) were the most common mutations in *EGFR* gene. Ten *EGFR* 19del subtypes were confirmed in 47 samples from 43 patients (Table 3). Deletions starting from E746 occurred in 38 samples (80.9%) and starting from L747 occurred in 9 ones (19.1%). DelE746_A750 was the most frequent subtype (33/47, 70.2%). For the patients receiving EGFR-TKIs treatment, no difference was found between patients with delE746_A750 and with other deletions in the objective response rate (ORR) (85% vs. 66.7%, *P* = 0.558) and no difference between patients with deletions starting from E746 and from L747 in ORR (81.8 vs. 75%, *P* > 0.999; Table 3, Additional file 1: Table S2). In 30 *EGFR*-mutated patients who received 1st generation EGFR TKIs treatment, 5 harbored T790M in 14 individuals with L858R, while 13 carried T790M in 16 individuals with 19Del (Fig. 1D). Patients with *EGFR* 19Del were more likely to acquire T790M mutation than those with L858R after they were treated with EGFR TKIs (*P* = 0.024).

**Table 3:** *EGFR* exon 19del subtypes in patients

No.	Subtypes	Number of altered samples	TKI response (total = 26)^**a**^	
			PR, N	PD, N
**1**	c.2235_2249del			
	p.Glu746_Ala750del	20	10	2
**2**	c.2236_2250del			
	p.Glu746_Ala750del	13	7	1
**3**	c.2236_2248delinsAGCC			
	p.Glu746_Ala750delinsSerPro	1		1
**4**	c.2236_2252delinsCT			
	p.Glu746_Thr751delinsLeu	1		
**5**	c.2236_2257delinsCTCT			
	p.Glu746_Pro753delinsLeuSer	1		
**6**	c.2237_2252delinsT			
	p.Glu746_Thr751delinsVal	1		
**7**	c.2237_2255delinsT			
	p.Glu746_Ser752delinsVal	1	1	
**8**	c.2239_2247del			
	p.Leu747_Glu749del	1		
**9**	c.2239_2256del			
	p.Leu747_Ser752del	1		
**10**	c.2240_2254del			
	p.Leu747_Thr751del	2	1	1
**11**	c.2240_2257del			
	p.Leu747_Pro753delinsSer	5	2	

^a^patients received the first-generation TKI treatment

**Fig. 3:**
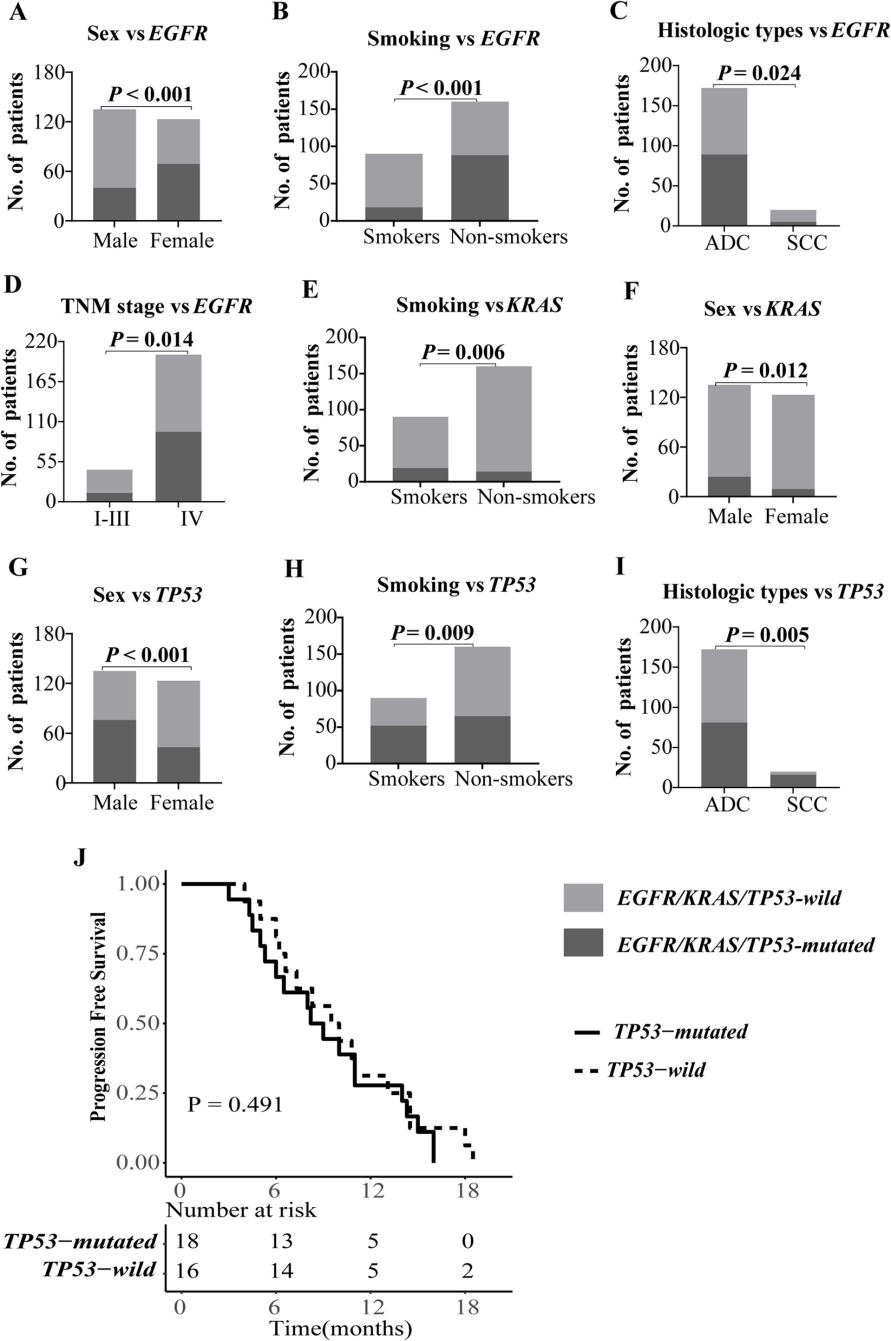
Analysis of mutations in *EGFR, KRAS* and *TP53*. **A**-**I** Correlation of patient clinical characteristics and the genetic alterations. **J** Progression free survival in 34 *EGFR* mutated patients with or without *TP53* mutation. “Number at risk” is the total number of participants in the study exposed to the risk of outcome events

The *EGFR* mutations were more frequent in females than males (56.1% vs. 29.6%, *P* < 0.001; Fig. 3A), in patients without smoking history than smokers (55% vs. 20%, *P* < 0.001; Fig. 3B), in ADC patients than SCC patients (51.7% vs. 25%, *P* < 0.001; Fig. 3C), and in IV stage patients than I-III stage patients (47.5% vs. 27.3%, *P* = 0.014; Fig. 3D).

### *KRAS* mutations

*KRAS* mutations were observed in 33 samples, most of which were in codons 12/13 (78.9%, 26/33) and codon 61 (15.2%, 5/33). Compared to codons 12/13, codon 61 mutations more tend to overlap with other frequent oncogenic alterations like *EGFR* L858R, 19Del, *ALK* rearrangement and *BRAF* V600E (60% vs. 3.85%, *P* = 0.008). No patient with mutations in codons 12/13 received targeted therapy, whereas three patients with *KRAS* codon 61 mutations showed stable disease or partial response after receiving TKI inhibitor ((Additional file 1: Table S3).

*KRAS* mutation status was found have association with smoking history (21.1% for smokers vs. 8.8% for non-smokers, *P* = 0.006; Fig. 3E), and gender (17.8% for males vs. 7.3% for females, *P* = 0.012; Fig. 3F). No association was found between *KRAS* mutation status and the histologic types, and clinical stage (Additional file 1: Table S4).

### *TP53* mutations

A total of 110 mutation types were identified in our cohort, most of which were observed in only one sample (Additional file 1: Table S1). The majority of these mutations were located within the p53 DNA-binding domains (exons 5–8), including R175H in 4 samples, R273L in 3 samples, etc.

The *TP53* mutation frequency in males was much higher than that in females (56.3% for vs. 35.0%, *P* < 0.001; Fig. 3G). The *TP53* mutated more frequency in patients with tobacco consumption than that in patients without smoking history (57.8% for vs. 40.6%, *P* = 0.009; Fig. 3H). The SCC had a higher frequency of *TP53* mutations compared to ADC individuals (80.0% vs. 47.1%, *P* = 0.005; Fig. 3I). No difference was found in the clinical stage between the *TP53* mutation (Additional file 1: Table S4).

Among the 107 samples with multiple gene mutations, concomitant *EGFR* and *TP53* mutations were found in 63 ones (58.9%). According to the follow-up data collected, only 34 *EGFR*-mutated patients were included in survival analysis. The median PFS was 8.6 months for *TP53* mutated patients and 9.8 months for *TP53* wild-type patients. No difference in PFS was found between *EGFR*-mutated patients with and without *TP53* mutations (*P* = 0.491; Fig. 3J).

### Genomic detection could improve clinical outcomes

**Fig. 4:**
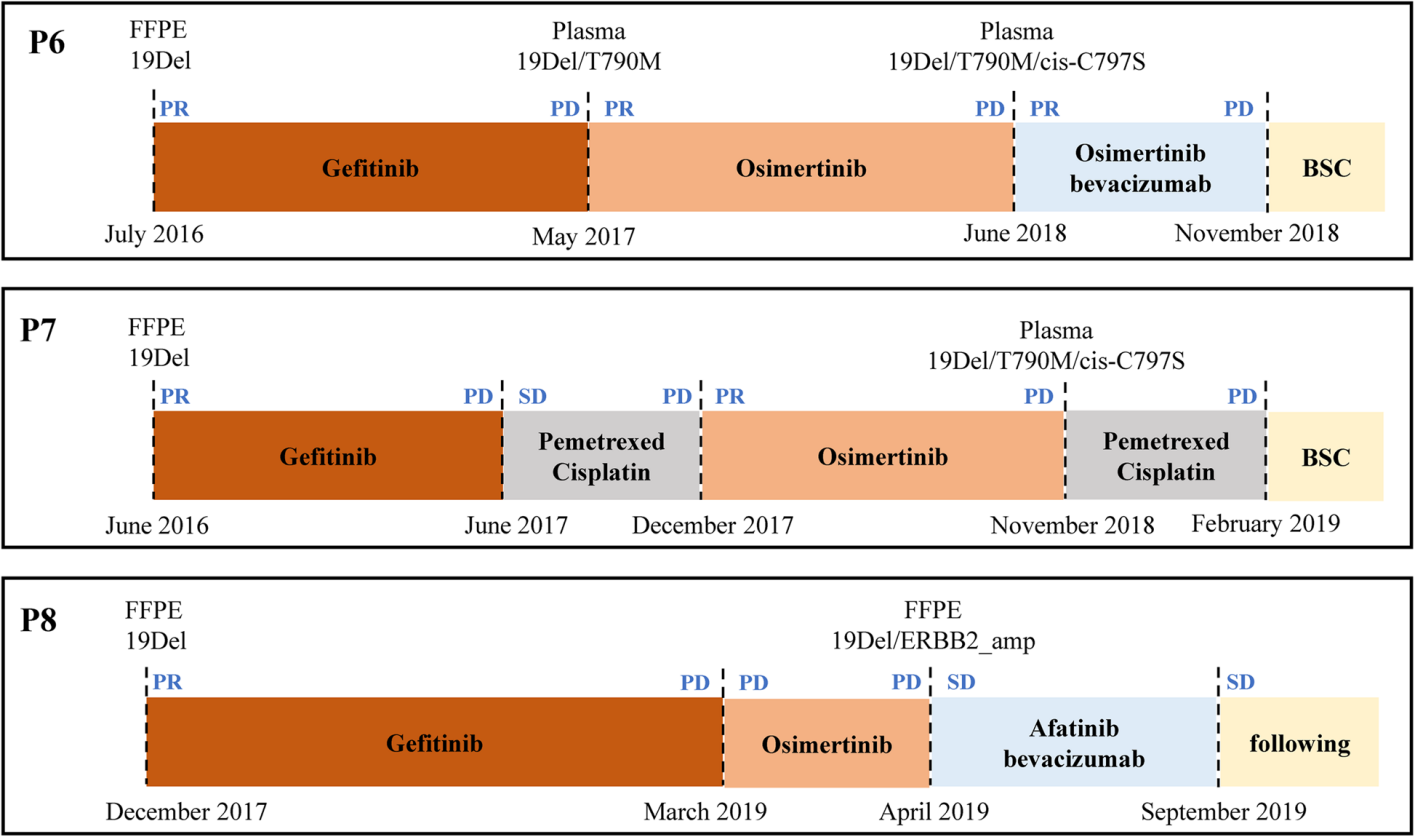
The treatment history and driver gene evolution for 3 patients who had acquired resistance to EGFR TKIs. BSC, best supportive care

In the current study, we described three patients (P6, P7, P8) with multiple gene detection results during the whole treatment history to see if the genomic characterization helped their clinical management (Fig. 4). The three patients all had acquired resistance to EGFR TKIs.

The first patient (P6) was a 63-year-old man diagnosed with lung adenocarcinoma with metastases to bone in July 2016. Because of the 19Del of *EGFR* identified by Amplification Refractory Mutation System (ARMS), he was treated with gefitinib and achieved partial responses (PR) for 11 months. However, progression disease (PD) was observed, and *EGFR* 19Del and acquired T790M were both identified by NGS in plasma in May 2017. Osimertinib was added and the patient responded for another 11 months. An acquired cis-C797S, which lead the drug resistant, besides with 19Del and T790M mutations were identified by NGS in plasma in June 2018. The patient was then treated with the combination of osimertinib and bevacizumab (4 weeks), under which the disease did not stop progressing (Fig. 4).

The second patient (P7) was a 67-year-old woman diagnosed with lung adenocarcinoma with metastases to multiple organs in June 2016. Due to the presence of 19Del by ARMS, she was treated with gefitinib, which resulted in clinically PR. About 11 months after therapy, CT scan showed increased lesions in both liver and lung. After 2 cycles of chemotherapy, the patient was treated with osimertinib and maintained PR for 11 months until December 2018. Then, *EGFR* cis-C797S combined with T790M and 19Del were identified by NGS in plasma DNA. This patient was treated with a standard chemo strategy with pemetrexed-cisplatin, which had a poor effect (Fig. 4).

The third patient (P8) was a 56-year-old woman diagnosed with lung adenocarcinoma with metastases to bone. ARMS revealed *EGFR* 19Del mutation in patient’s surgical specimen. She was treated with gefitinib and achieved PR for 16 months until PD. Without any genetic test, this patient was treated with Osimertinib, but no response was observed. Later, the NGS resulted from plasma DNA revealed an acquired *ERBB2* amplification in conjunction with 19Del. The patient was then treated with a combination of afatinib, bevacizumab, and radiotherapy, which resulted in clinically stable disease (SD) at the last follow-up on Sep 1, 2019 (Fig. 4).

### Association of treatment selections with patient survival

**Fig. 5:**
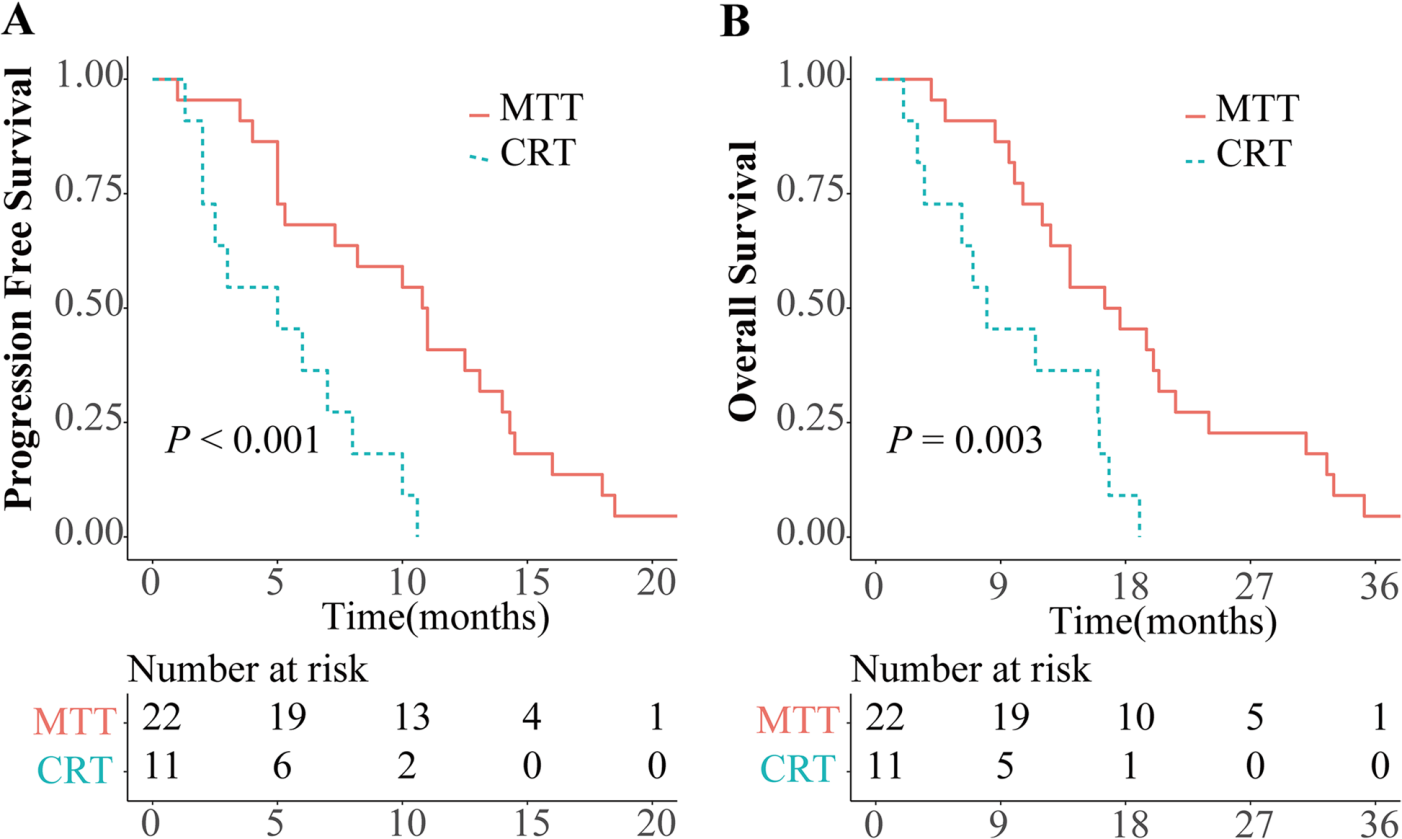
Kaplan–Meier curves for progression-free survival (PFS) (**A**) and overall survival (OS) (**B**) of all 33 patients. MTT, molecular targeted therapy; CRT, chemoradiotherapy

The median PFS and median OS of patients treated with EGFR TKIs alone was 10.9 and 17.1 months, which showed significantly longer than those of patients (PFS: 5.0; OS: 8.0 months) treated with chemoradiotherapy (*P* < 0.001 and *P* = 0.003, respectively) (Fig. 5).

## Discussion

Most lung cancer patients have no obvious specific symptoms at the beginning of the disease [11]. Older patients are usually with the high prevalence of comorbid disease that makes lung cancer more difficult to be detected at an early stage. In this study, lung cancer patients older than 55 years were enrolled, the majority of which were in stage IV. Comprehensive genomic spectrum on 267 lung cancer specimens from 258 patients aged more than 55 years showed a heterogeneity in the current study. The rare *MET* 14 exon mutations, as well as some common druggable genes like *EGFR*, *KRAS*, and *ALK* were detected in cases that sampled before any treatment. *ERBB2* amplification, a bypass mechanism of resistance to EGFR TKIs, besides with the common *EGFR* T790M resistance mutation were identified in cases that sampled after EGFR TKIs treatment. We explored the genetic status of patients with both the FFPE and plasma samples, and no difference of gene alteration was found between the FFPE and plasma samples except for *EGFR*. The *EGFR* mutation was also more frequent in stage IV compared to stage I-III patients. For these two situations, *EGFR* T790M which occurred during the tumor progression and medical treatment may be the major cause. Some samples for stage IV patients and some plasma samples were collected after a period of treatment, which resulted an increase of *EGFR* T790M. When we compare the samples collected before treatment, no difference of gene alteration was found between the FFPE and plasma samples. Similar to the reported [12], *EGFR* mutations were more likely to be occurred in non-smokers, female and ADC patients. Besides, we found that *EGFR* was mutated at a much higher frequency in our cohort than in Caucasians when we compared our data to the TCGA dataset. Studies showed that female sex, adenocarcinoma histology, never-smoking status, and Asian ethnicity are considered the most important factors associated with *EGFR* mutation [13], and mutations of *KRAS* and *TP53* were more frequent in males and smokers other than females and non-smokers [14, 15]. These biases were all validated in our results, which suggested that the characteristics of *EGFR, KRAS* and *TP53* in our study were similar with others cohort.

*EGFR* is a transmembrane tyrosine kinase receptor which activates Jak, PI3K, ROS, and RAS pathways leading to cell survival [16]. The presence of the main activating mutations, including L858R mutation and the exon 19Del is associated with sensitivity to *EGFR*-TKIs [17]. Studies showed that *EGFR* 19Del was associated with better outcomes in treatments with EGFR-TKIs than L858R mutation [18]. Some investigators have explored the structures of 19Del and L858R and suggested different binding affinity of TKIs [19]. Here, we reported a higher prevalence of T790M mutation in 19Del cohort than that in L858R cohort, consistent with previous research [20], this may be a mechanism of different outcomes of these two alteration subtypes. It was reported that the 19Del subtypes could also influence different clinical outcomes to EGFR-TKIs [21], while others found no significant differences between patients with different subtypes [22]. In this study, no significant differences in ORR between different *EGFR* 19Del subtypes. However, due to limited sample size, it was not reasonable to definitively conclude which subtype had the most important influence. The T790M mutation in the ATP-binding site of *EGFR* is the most common mechanism of resistance to 1st and 2nd generation EGFR TKIs [23]. The role of methionine (M) mutated from threonine (T) acts as a “gatekeeper” residue causing steric hindrance thus decreasing hydrophilicity and preventing tyrosine kinase binding [24]. Moreover, T790M mutation increases the affinity for ATP in EGFR kinase causes drug resistance [25]. Other mechanism for resistance like *EGFR* C797S mutation [26], and *ERBB2* amplification [23], were also identified in our studies. We presented the clinical procedure of 3 subjects and demonstrated the value of genomic detection for helping clinicians to distinguish specific resistance mechanisms in each patient and make personalized medicine strategies. Genomic evolution of tumor is the major obstacle in long-term response to TKI treatment. So that it is necessary for lung cancer patients, especially the old patients with high heterogeneity to investigate their genomic profile during their medical treatment.

*KRAS* mutations, with codons 12 and 13 mutations being the most frequent, are oncogenic drivers in lung cancer [27]. These mutations cause constitutive activation of the RAS signaling pathway, and further activate several downstream signaling effectors such as the canonical Raf-MEK-ERK, the PI3K-AKT-mTOR, RalGDS-RalA/B pathways and the TIAM1-RAC1 pathway [28]. The frequency of *KRAS* codon 61 mutation in our study was higher than the previously reported rate of TCGA cohorts (1%) that included the large number of Caucasian patients, and similar to the frequency of East Asian (13%) [29]. This discrepancy may partially attribute to the difference of ethnicity and age. Some studies have not found *KRAS* codon 61 mutations in the young patients with NSCLC [30, 31], and indicated that the incidence of codon 61 mutation was more frequent in the older patients with colorectal cancer [32]. Unlike G-C or G-T mutating more in patients with smoking history [33], all codon 61 mutations identified in our study were transversion mutation (A-T) and most of which were occurred in non-smokers. Overall, such cancers with codon 61 mutations may be caused by the accumulation of years other than environment tobacco exposure.

The prognostic value of *TP53* mutations in *EGFR*-mutated lung cancer is still a controversial issue. Some researchers suggested that *TP53* mutations were associated with shorter survival in *EGFR*-mutated patients [34]. However, a couple of studies have failed to demonstrate this association between survival and *TP53* mutations [35, 36], which was in line with our findings that harboring a *TP53* mutation did not significantly affect PFS in *EGFR*-mutated patients. It might be speculated that *TP53* mutation as a prognostic marker is unclear in lung cancer patients with *EGFR* mutation, and the *TP53* status cannot be used to select treatment for *EGFR*-mutated patients until now. The influence of *TP53* mutational status on *EGFR*-mutated patients with lung cancer is required to clarify with larger datasets.

Molecular targeted therapies have been developed to specifically block cancer growth [37]. Especially, EGFR TKIs are demonstrated to have ability to prolong survival time of *EGFR*-mutated patients with lung carcinoma [38, 39]. In concordance with these reports, targeted therapy is associated with better PFS and OS compared with conventional chemoradiotherapy in the current study. Previous studies showed that molecular targeted therapy plus chemotherapy improved PFS compared with targeted therapy alone lung cancer patients [40, 41]. However, we did not get such data because of the relatively small sample size that treated with TKIs plus chemotherapy. For *EGFR* mutated elderly patients with poor performance status, EGFR TKIs, maybe a recommended option that could improve survival time compared with chemoradiotherapy alone.

## Conclusions

In this study, aged patients with lung cancer showed some specific gene alterations, such as a relative high proportion *KRAS* codon 61 in the *KRAS* mutated patients, and the various mechanism of EGFR TKIs resistance. The use of NGS technology can help clinicians making personalized medicine strategies, especially distinguishing specific resistance mechanisms in each patient. Besides, these old patients in our cohort were benefit from the targeted treatment other than chemoradiotherapy alone. However, further studies are required to confirm these findings and a much larger sample size is needed to analyze the association of treatment selections with patient survival.

## Methods

### Patients and samples

We retrospectively analyzed 267 samples including 118 FFPE tissues and 149 plasma specimens from 258 lung carcinoma patients, collected in Central Hospital of Guangdong Nongken during December 2016 and December 2020. Based on the individual wishes of patients and their families, the patients were treated according to Chinese Medical Association guidelines for clinical diagnosis and treatment of lung cancer [42, 43] or the National Comprehensive Cancer Network guidelines [44, 45]. Written Informed consent was provided by all patients before testing. All the participants, samples and data involved in our study have been performed in accordance with the Declaration of Helsinki, and approved by the Ethics Committee of Central Hospital of Guangdong Nongken (No.2018001).

About 10 mL peripheral blood was collected from each patient into EDTA-containing tubes or cell-Free DNA BCT® tube (Streck Inc., Omaha, USA). Plasma was isolated using a double centrifugation protocol by centrifugation at 1600 g for 10 min, followed by 16,000 g for 10 min, and stored at − 80 °C until subsequent analysis. Tumor tissues were fixed in 10% neutral buffered formalin overnight and were routinely embedded in paraffin, with conformation by the pathologists for diagnosis and tumor purity.

### DNA extraction

Genomic DNA was extracted from unstained 10-μm thick FFPE sections with tumor content more than 10% using the QIAamp DNA FFPE Tissue Kit (Qiagen, Hilden, Germany) following the manufacturer’s instructions. Circulating cell-free DNA (cfDNA) was recovered from the plasma samples using the QIAamp Circulating Nucleic Acid kit (Qiagen). After extraction, DNA quality was evaluated by 1% agarose gel electrophoresis and the concentration of all samples was quantified using the Qubit dsDNA HS Assay kit (Thermo Fisher Scientific, Waltham, MA, USA) with a Qubit 3.0 Fluorometer.

### Next generation sequencing and data analysis

A probe-based targeted NGS was used for library generating with an OncoAim® Lung cancer targeting gene detection kit (Singlera Genomics, Inc., Shanghai, China), of which the gene panel included all exons of 12 genes (*ALK*, *BRAF*, *EGFR*, *ERBB2*, *FGFR1*, *KRAS*, *MET*, *NRAS*, *PIK3CA*, *RET*, *ROS1*, *TP53*) involved in tumorigenesis, and potential gene rearrangement/fusion of *ALK*, *ROS1* and *RET*. According to the kit protocol, the input of FFPE DNA and cfDNA for library preparation was 50 ng and 30 ng, respectively. Accordingly, FFPE DNA were sheared to about 250 bp with restriction enzyme before library construction. The cfDNA libraries were constructed with 12 bp unique molecular identifier (UMI) sequences to distinguish PCR-duplicated fragments. After the end repair, A-tailing, and adapter ligation, target capture with probes supplied in the kit was performed according to manufacturer’s specifications. The library product was sequenced using 150 bp paired-end runs on the NextSeq 500 (Illumina, Inc., San Diego, CA, USA), with an average sequencing depth of FFPE library and cfDNA was 1000X and 20,000X, separately.

Sequencing data were processed following the guideline of the OncoAim® kit (Singlera), which was designed to simultaneously detect single nucleotide variations (SNV), short insertions and deletions (InDels), copy number variations (CNV) and gene rearrangements. Briefly, sequencing reads were quality-filtered with FastQC (version 0.9.5, Babraham Bioinformatics, Cambridge, UK), and assembled and aligned against the reference genome hg19/GRCh37 by the Burrow-Wheeler Aligner algorithm (https://github.com/lh3/bwa; version 0.7.12-r1039; Dec 2015). Unique reads derived from GATK were used for variant calling. Insertions and deletions in sequence alignment files were left-aligned using Freebayes (https://github.com/ekg/freebayes). For the NGS data sequenced from cfDNA samples, the UMI was used as a variants filter. The minimum confidence threshold for variant and insertion/deletion (indel) calling was set to 0.001 (0.1%) for cfDNA samples and 0.02% (2%) for FFPE samples.

### Statistical analysis

Statistical analysis was performed using R version 4.0.4 (R Development Core Team, 2019) and SPSS 22.0 software (SPSS, Chicago, USA). The chi-square test or Fisher’s test was used to analyze the association of mutational status detected for the first sampling in our study with clinical features. The Kaplan-Meier method with a log-rank test was used to estimate the median progression free survival (PFS) and median overall survival (OS). *P* value < 0.05 was considered statistically significant.

## Supplementary Information

**Additional file 1: Table S1-S4.**

**Additional file 2: Figure S1.**

## Data Availability

All data generated or analyzed during this study are included within the article.

## Abbreviations

CT: Computed tomography
PET: Positron emission tomography
NGS: Next generation sequencing
FFPE: Formalin-fixed paraffin-embedded
cfDNA: Circulating cell-free DNA
SNV: Single nucleotide variations
InDels: Short insertions and deletions
CNV: Copy number variations
PFS: Progression free survival
OS: Overall survival
TKIs: Tyrosine kinase inhibitors
ADC: Adenocarcinoma
SCC: Squamous cell carcinoma
ASC: Adenosquamous carcinoma
19Del: Exon 19 deletions
ARMS: Amplification Refractory Mutation System
PR: Partial responses
PD: Progression disease
SD: Stable disease
BSC: Best supportive care
MTT: Molecular targeted therapy
CRT: Chemoradiotherapy
