# Supplementary figures and images for: Investigating the genomic alteration improved the clinical outcome of aged patients with lung carcinoma
Source: BMC Genomics. 2022 Jan 15;23:55. doi: 10.1186/s12864-021-08289-4 (PMC8760649; doi:10.1186/s12864-021-08289-4)

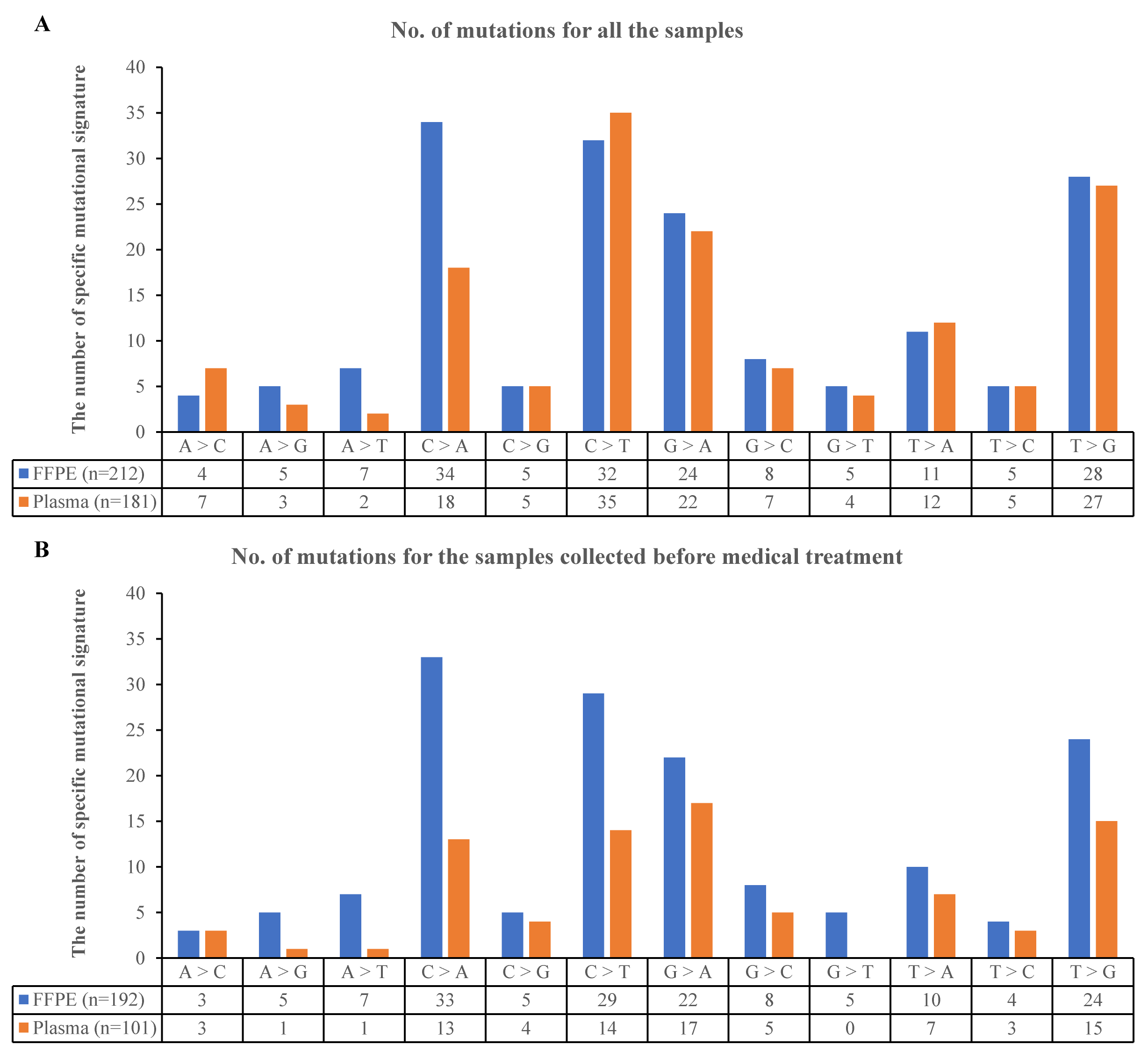

Supplement: Supplementary file 2 — Additional file 2: Figure S1. [file 12864_2021_8289_MOESM2_ESM.tif]
